# Supplementary material for: Causal interplay between lactose intolerance and gut microbiota: a combined bidirectional Mendelian randomization and in vivo validation study
Source: Front Nutr. 2026 Jun 1;13:1803337. doi: 10.3389/fnut.2026.1803337 (PMC13265576; doi:10.3389/fnut.2026.1803337)
Supplement: Supplementary file 1 [file Data_Sheet_1.zip › supplementary materials/Forward/leave one out cross validation/ebi-a-GCST90027619.finngen_R12_E4_LACTONAS.pdf]

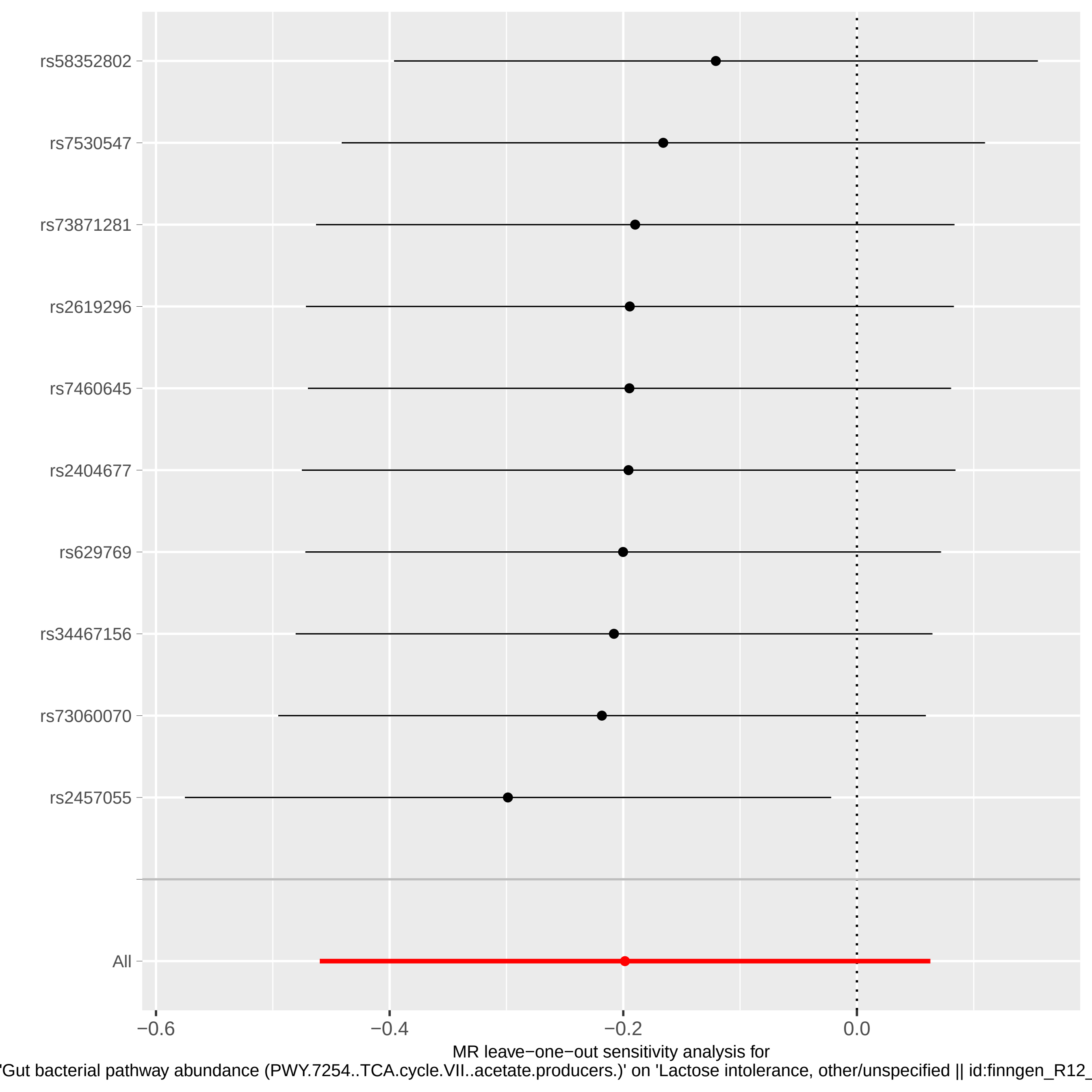
rs58352802
rs7530547
rs73871281
rs2619296
rs7460645
rs2404677
rs629769
rs34467156
rs73060070
rs2457055
All
−0.6
−0.4
−0.2
0.0
MR leave−one−out sensitivity analysis for
'Gut bacterial pathway abundance (PWY.7254..TCA.cycle.VII..acetate.producers.)' on 'Lactose intolerance, other/unspecified || id:finngen_R12_
